# Fatal Systemic Air Embolism in a Neonate after Cardiopulmonary Resuscitation

**Published:** 2015-01-01

**Authors:** Abid Quddus Qazi, Zulfiqar Ali Haider, Yawar Najam

**Affiliations:** 1 Shaukat Khanum Memorial Cancer Hospital and Research Centre, Lahore, Pakistan.; 2 Shifa International Hospital, Islamabad, Pakistan.

**Dear Sir,**

Vascular air embolism has been known since early 19th century.[1] Many cases have been reported specially in paediatric patients under the age of 3 months and those receiving intensive care. Amongst the many causes proposed, vigorous cardiopulmonary resuscitation and intravenous infusion/pump have long been suspected. Here we would like to share such a case.

A neonate, born at term, weighing 2.5 kg, presented to accident and emergency department at 5 hours of age with respiratory distress since birth. He was born through a normal delivery in another hospital and was diagnosed to have left congenital diaphragmatic hernia. After initial endotracheal intubation, resuscitation and stabilization, he underwent an uneventful repair of a small defect of diaphragm on 2nd day of life. No chest drain was inserted at operation to keep a small volume of pneumothorax to splint left hypoplastic lung. Subsequently he was extubated after 24 hours of surgery (day 3 of life) and once the gastric aspirate cleared, started on enteral feeds via nasogastric tube. On day six of life, in the morning, he was self ventilating on low flow oxygen, tolerating enteral feeds via nasogastric tube at 50% of requirement. In addition he was receiving intravenous fluids via a 24G peripheral cannula using an infusion pump (I-Med® infusion pump). A chest X-ray showed that most of the pneumothorax had reabsorbed. However in the same evening he suddenly collapsed with cyanosis, bradycardia and severe hypoxemia. Cardiopulmonary resuscitation was carried out with endotracheal intubation but declared unsuccessful after 30 minutes. A chest X-ray was taken in search of aetiology of sudden collapse after 30 minutes of resuscitation. This revealed significant volume of air in the right atrium, systemic and pulmonary vasculature (Fig.1).

**Figure:**
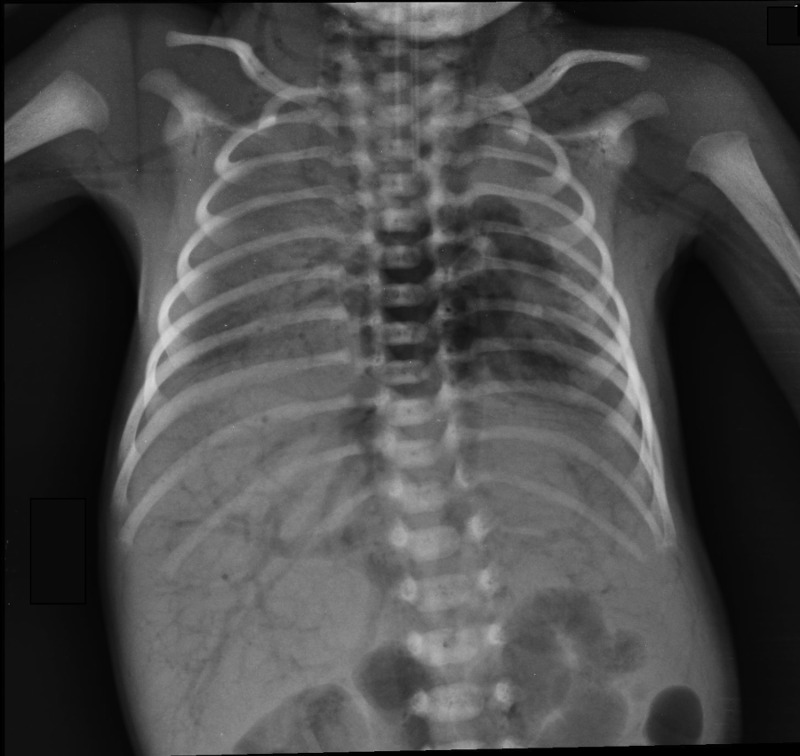
Figure 1:Massive systemic air embolism, delineating air contrast in both venous and arterial vasculature and a large volume in all four chambers of the heart.

There was no obvious cause of cardiovascular collapse in this baby; however with the background of diaphragmatic hernia repair, gastro-oesophageal reflux could be a possibility. As autopsy is not in the practice of medicine in Pakistan, many questions remain unanswered. However one can argue possible causes of systemic air embolism in this patient. The possibility of this via a 24G peripheral venous cannula through automated infusion pump does not seem a practical possibility in the wake of massive air embolism delineating the entire systemic and pulmonary vasculature. The automated pumps have built in safety mechanism to stop functioning in the presence of air bubbles and bleeps alarm but there have been cases reported where alarm did not go off but the patient collapsed and could not be revived and autopsy showed air in the cardiac chambers.[2] However the volume of air infused was very small and only possible to identify on cardiac echo. In our patient the other possible cause was vigorous chest compressions and assisted ventilation during resuscitation. Air embolism after resuscitation has been often reported in literature but this is usually observed in cerebral vasculature.[3,4] Systemic air embolism has been reported in premature infants on artificial ventilation as a result of baro-trauma as well as after necrotizing enterocolitis.[5,6] There is only a single case report in literature with systemic air embolism in a premature infant who collapsed suddenly due to a clot in inferior vena cava.[6] It is hypothesized that there is disruption of pulmonary vasculature during resuscitation, which leads to air entry from the alveoli, possibly because of frail tissues/vasculature in newborns . However, the role of pneumothorax is not clear. There are reports of systemic air embolism associated with assisted ventilation in premature infants.[7] An early suspicion of diagnosis, urgent echo at the time of collapse and initiation of treatment for systemic air embolism may play a role in saving life in such a scenario. Medical equipment should also be critically checked at regular intervals by the relevant electromedical department for any malfunction. These possible explanations may be kept in mind while investigating cause of demise in similar situations.

## Footnotes

**Source of Support:** Nil

**Conflict of Interest:** None declared

